# Evaluation of 4D CT acquisition methods designed to reduce artifacts

**DOI:** 10.1120/jacmp.v16i2.4949

**Published:** 2015-03-08

**Authors:** Sarah J. Castillo, Richard Castillo, Edward Castillo, Tinsu Pan, Geoffrey Ibbott, Peter Balter, Brian Hobbs, Thomas Guerrero

**Affiliations:** ^1^ Department of Radiation Oncology The University of Texas Medical Branch Galveston TX; ^2^ Department of Radiation Oncology Beaumont Health System Royal Oak MI; ^3^ Department of Computational and Applied Mathematics Rice University Houston TX; ^4^ Division of Diagnostic Imaging, Department of Imaging Physics The University of Texas M.D. Anderson Cancer Center Houston TX; ^5^ Division of Radiation Oncology, Department of Radiation Physics The University of Texas M.D. Anderson Cancer Center Houston TX; ^6^ Division of Quantitative Sciences, Department of Biostatistics The University of Texas M.D. Anderson Cancer Center Houston TX USA

**Keywords:** 4D CT, artifacts, radiation therapy simulation, correlation

## Abstract

Four‐dimensional computed tomography (4D CT) is used to account for respiratory motion in radiation treatment planning, but artifacts resulting from the acquisition and postprocessing limit its accuracy. We investigated the efficacy of three experimental 4D CT acquisition methods to reduce artifacts in a prospective institutional review board approved study. Eighteen thoracic patients scheduled to undergo radiation therapy received standard clinical 4D CT scans followed by each of the alternative 4D CT acquisitions: 1) data oversampling, 2) beam gating with breathing irregularities, and 3) rescanning the clinical acquisition acquired during irregular breathing. Relative values of a validated correlation‐based artifact metric (CM) determined the best acquisition method per patient. Each 4D CT was processed by an extended phase sorting approach that optimizes the quantitative artifact metric (CM sorting). The clinical acquisitions were also postprocessed by phase sorting for artifact comparison of our current clinical implementation with the experimental methods. The oversampling acquisition achieved the lowest artifact presence among all acquisitions, achieving a 27% reduction from the current clinical 4D CT implementation (95% confidence interval=34−20). The rescan method presented a significantly higher artifact presence from the clinical acquisition (37%; p<0.002), the gating acquisition (26%; p<0.005), and the oversampling acquisition (31%; p<0.001), while the data lacked evidence of a significant difference between the clinical, gating, and oversampling methods. The oversampling acquisition reduced artifact presence from the current clinical 4D CT implementation to the largest degree and provided the simplest and most reproducible implementation. The rescan acquisition increased artifact presence significantly, compared to all acquisitions, and suffered from combination of data from independent scans over which large internal anatomic shifts occurred.

PACS numbers: 87.57.C‐, 87.57.cp, 87.57.Q‐, 87.55.Gh

## I. INTRODUCTION

Four‐dimensional computed tomography (4D CT) is routinely employed as an integral part of radiation therapy simulation when there is a need to account for respiratory motion.^1^, ^2^, ^3^, ^4^, ^5^ Four‐dimensional CT correlates the image acquisition with the patient's breathing, resulting in a series of 3D image volumes that represent the entire breathing cycle.^6^, ^7^ This method enables a more accurate treatment delivery by limiting the uncertainty in the target cancer location as it translates and/or deforms with respiratory motion.^3^, ^8^, ^9^, ^10^, ^11^, ^12^, ^13^, ^14^, ^15^ However, the ability of 4D CT to limit uncertainties associated with anatomic position depends on image quality.

Artifacts present as artificial anatomic spatial distributions and cause uncertainty of the true anatomic position and configuration with breathing, potentially leading to errors in treatment planning delineation and targeting. Additionally, 4D CT artifacts have been demonstrated to affect emerging applications, such as lung function imaging derived from CT.^16^, ^17^, ^18^, ^19^, ^20^ In addition to artifacts common in diagnostic CT, 4D CT images are subject to artifacts resulting from aspects of the 4D acquisition and processing, often caused by irregular breathing.^1^, ^17^, ^18^, ^21^, ^22^, ^23^, ^24^, ^25^ While breathing irregularities may introduce appreciable artifacts into the 4D CT, the current clinical strategy is to use the low‐quality dataset for treatment planning or to reacquire the 4D CT scan. If the reacquired 4D CT demonstrates appreciable artifacts, free breathing helical‐acquired CT images are used for aid in treatment planning; use of these images was standard practice prior to the introduction of 4D CT methods and does not account for respiratory motion.

Several researchers have demonstrated methods to reduce 4D CT artifacts by reducing the amount of data associated with breathing irregularities. Langner and Keall^26^, ^27^, ^28^ compared sorting methods to reduce respiratory motion artifacts in a retrospective simulation study.

A model simulated CT images when a patient respiratory signal fell within tolerance to a reference respiratory trace and only those images were used in the sorting process. By simulating images only when the respiratory waveform was within a tolerance of the reference signal, a higher quality 4D CT was produced compared to retrospective phase sorting using all images.^26^, ^27^, ^28^

Pan et al.^(24)^ reduced cine 4D CT artifacts by locating data acquired during irregular breathing and disabling its use in phase sorting. Sample coronal and sagittal views visually demonstrated improved image quality when these irregular portions were excluded; however, this was only applied to one region of the scan extent.

Keall et al.^(23)^ explored the potential of artifact reduction through prospective gating by halting image acquisition for a breathing irregularity. Coronal images of a phantom were acquired both during a breathing irregularity, and then using beam gating during the irregularity. The image acquired using the gating was found by visual comparison to exhibit improved quality compared with the image that was not gated.

Despite these promising results, to the best of our knowledge, methods to prospectively reduce 4D CT artifacts by altering the acquisition have not been attempted in a clinical setting. In the present study, we evaluated the ability to reduce artifacts for any given breathing pattern using three experimental 4D CT acquisition methods: 1) acquiring more images, 2) gating the X‐ray beam with breathing irregularities, and 3) reacquiring images associated with breathing irregularities. We enrolled thoracic cancer patients in an IRB‐approved protocol so that they would receive each experimental scan in addition to the clinical 4D CT scan. This allowed determination of the potential for improving 4D CT image quality in a clinical setting and in a relatively simple manner. Unlike prior studies, the methods used in this study focused on acquisition modification rather than retrospective analysis.

## II. MATERIALS AND METHODS

### A. Patients

With approval from the M.D. Anderson Cancer Center Institutional Review Board (2011‐0631), our study included 18 patients scheduled to receive thoracic radiation therapy, 8 women and 10 men. Clinical diagnoses included non‐small cell lung cancer (n=12), esophageal cancer (n=4), and mesothelioma (n=2). The mean (± standard deviation [SD]) age of study participants was 66.3±10.1 yrs. Each patient received a standard 4D CT simulation, immediately followed by each of the three experimental acquisition methods.

Patient enrollment in the study depended on certain inclusion and exclusion criteria. Patients had to be at least 18 years of age, eligible to receive radiation therapy at our institution, pathologically diagnosed with a primary thoracic malignancy, and they had to sign consent for study participation. Patients who were pregnant or had an implanted electronic device such as a pacemaker were not eligible to enroll in this study. Patients with a history of claustrophobia were not considered. No breathing information was known prior to enrollment or scan acquisition.

### B. 4D CT

All 4D CT images were acquired in cine mode on a GE Discovery ST PET/CT scanner (GE Medical Systems, Waukesha, WI) with a 500 ms tube rotation time; the CT component is an 8‐slice LightSpeed CT. A real‐time processing monitor (RPM; Varian Associates, Palo Alto, CA) served as an external surrogate for organ motion, which provided a respiratory trace of relative abdominal height versus time.

Cine 4D CT acquires images in beam‐width steps that correspond to fixed couch positions to cover the superior–inferior scan extent. Acquiring images for at least 1 breathing cycle per couch position ensures data sufficiency; therefore, the acquisition time per couch position (cine duration) was based on the patient's average breathing cycle plus 1 s for all but the oversampling method. This yields several multislice image segments (8×2.5 mm axial images) per couch position. Images from all acquisition methods were reconstructed using 360° of data every 350 ms following the first 500 ms rotation at each couch position. Acquisitions were obtained at 120 kVp, with a 100 mA tube current for clinical acquisitions and a dose‐sparing 50 mA for experimental acquisitions. Each acquisition was processed to yield a final 4D CT with a set of ten 3D CT volumes, each representing a component breathing phase.

### C. Oversampling method

For the oversampling method (OM), the cine duration was increased to capture at least 2 breathing cycles per couch position instead of the 1 breathing cycle per couch position the clinical acquisition captures. This was implemented by altering the manufacturer's internal data acquisition constraint from 3000 to 6000 images to allow for extended cine durations. When possible, the scan extent was reduced to minimize the dose the patient received.

### D. Gating method

To implement the gating method (GM), we monitored the respiratory trace in real time to facilitate manual X‐ray beam gating when an apparent breathing irregularity occurred, and subsequently restarted the X‐ray beam when regular breathing returned. An observer (SJC) identified breathing irregularities manually for all gating acquisitions in real time. The respiratory trace was then analyzed retrospectively for breathing irregularities using the method outlined in the Rescan Method section (Materials & Methods section E below). Manually stopping the X‐ray beam caused reacquisition of 1 cine duration at the interrupted couch position to ensure a full breathing cycle of data was acquired before proceeding to the next couch position; this yielded a full unsorted 4D CT image set plus gated sections. When possible, the scan extent was reduced to minimize the dose the patient received.

### E. Rescan method

For the rescan method (RM), image segments were reacquired at locations in which breathing irregularities occurred during the clinical acquisition. Implementation of the RM involved postprocessing the clinical 4D CT respiratory trace while the patient remained on the table to identify couch positions that were to be reacquired. Only the identified couch positions were rescanned and replaced in the clinical 4D CT for the final RM image set.

**Figure 1:**
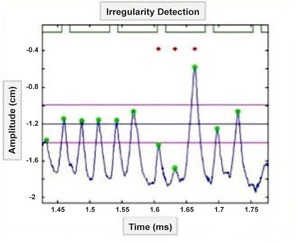
Breathing irregularity identification. Patient respiratory trace (blue), beam‐on signal (green lines), T0% phases (green circles). T0% mean (middle horizontal dark blue line), T0% SD (outer pink horizontal lines), T0% irregular phases (red stars).

To identify breathing irregularities for repeat imaging, we defined a tolerance range that was based on the mean and standard deviation of the 10 phase amplitudes across the scan extent. A breathing phase was considered irregular when the associated amplitude fell out of the tolerance range defined by the mean phase amplitude ±1 SD. A couch position warranted repeat if ≥30% of the location was associated with irregularities. This threshold was chosen based on an expected minimal 30% artifact reduction if three of ten image segments were replaced. An example of an irregular T0% phase in a patient respiratory trace is shown in Fig. 1. Breathing irregularities were quantified retrospectively for each of the four 4D CT acquisition techniques using this method of identification. A percentage of breathing irregularities in the respiratory trace was calculated with respect to the total number of breathing phases. All respiratory traces acquired in the study were analyzed for breathing irregularities using custom MATLAB software (The MathWorks, Inc., Natick, MA).

### F. Scan order

All 4D CT methods were acquired within the clinical time slot of an hour. As the clinical scan provided the image set used for treatment planning, the clinical acquisition always preceded the experimental scans. Given the RM combines data from the clinical scan, the RM was always acquired immediately following the clinical acquisition. The OM and GM acquisition order were alternated following the RM acquisition in an attempt to offset a potential bias in the results caused by an identical scan order for each patient. The RPM reflective box remained in the same position through the acquisitions for consistency.

### G. Postprocessing

All acquisition methods were postprocessed by a quantitative image correlation‐based sorting method. In addition, the clinical acquisitions were postprocessed with the current clinical standard of phase sorting. Each processing method begins with the user identifying peak inhalation locations on the respiratory trace. The breathing phases are then linearly defined over the breathing period, T, in increments of 10% (i.e., T0%, T10% … T90%) where T0% is the user‐defined peak inhalation time. At each couch position, an image segment per breathing phase is identified to represent that breathing state at that anatomic position.

**Figure 2:**
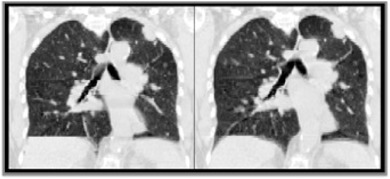
Phase‐sorted (left) and CM‐sorted (right) clinical acquisition sample coronal views.

In phase sorting, the image segment that occurred nearest in time to a phase definition is chosen to represent that breathing state. The correlation‐based sorting method first starts with this phase sorting method of identifying image segments; but the three nearest image segments to a phase definition are binned as opposed to one image segment. Therefore there are at least three image choices per breathing phase per couch position; this allowed retention of breathing information with the potential for artifact reduction in available data. We then incorporated a previously described metric of 4D CT artifact evaluation^(29)^ that is based on correlation coefficients across couch positions, termed CM. The absolute value of the sum of CM values across the scan extent was minimized using the shortest path Dijkstra's algorithm.^30^, ^31^ The image segments chosen to represent each breathing phase in the 4D CT were the image segments that achieved the optimal minimization of the CM values. We have found this sorting method, termed CM sorting, to improve artifact presence from phase sorting by 24% mean CM values (Fig. 2). This is consistent with previous findings from correlation‐based image processing techniques.^31^, ^32^, ^33^, ^34^ All processing was performed using custom MATLAB software.

### H. Effective dose estimates

The effective dose delivered to each patient per acquisition method was estimated using the CTDI method.^(35)^ The estimate was based on a measured ${\text{CTDI}}_{\text{vol}}$ of 50 mGy for a cine duration of 5.6 s and a tube current of 100 mA with a k‐factor for an adult chest of 0.014 mSv/mGycm.^(24)^ All experimental acquisition tube currents and scan extents were adjusted to match that from the clinical acquisition for equal comparison among acquisition methods.

### I. Analysis

Statistical analysis of the mean CM values included a one‐way mixed ANOVA model with heteroscedastic variance by acquisition method. An F‐test was used to test for association with acquisition method. Pairwise comparisons among the four acquisition methods used simultaneous inference with Tukey's method. The familywise error rate was controlled at the 0.05 significance level. Tukey's adjusted, two‐sided p‐values are reported. Interval estimation of the percentage reduction in CM values averaged over acquisition method and phase is provided using two‐side 95% confidence interval.

Estimates for the incidence of breathing irregularities among the four acquisition methods was calculated using maximum likelihood estimation of the log‐odds using a logistic ANOVA model with interval estimates derived from Wald standard errors.

## III. RESULTS

### A. Summary statistics

The mean percentage of breathing irregularities present during image acquisition for all 72 4D CT scans was 28.0%±7.7%. Respiratory trace parameters derived over the scan extent include a mean displacement from inhale to exhale of 0.98±0.41 cm, and an average breathing period of 4.34±1.3 seconds. The mean percentage of the reacquired scan extent for the RM was 40.3%±10.0%; a mean of 37.8%±18.1% of the images were reacquired in the lung. A mean of 28.4%±11.2% of breathing irregularities occurred during the reacquisition of images for the RM. A mean of 54.6%±12.1% of breathing irregularities were not gated during the GM image acquisition, a 26.7% reduction from the clinical acquired irregularities. The GM led to a 66.7%±39.2% increase in acquisition time compared with the clinical 4D CT acquisition time; this was due to the couch repetition for each beam stop and the time intervals between X‐ray beam on during irregular breathing. Automatic X‐ray beam control and acquisition 1 cine duration per couch position would decrease this scan duration. Despite the duration increase, all clinical and experimental 4D CT scans were acquired within the standard clinical 60 min time slot.

### B. Effective dose estimates

**Figure 3:**
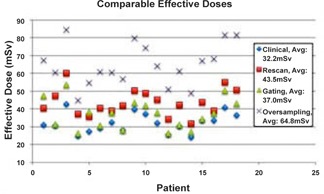
Estimated effective doses. All doses estimated using the clinical tube current and scan extent. The mean of the effective dose estimates per acquisition method are shown in the legend.

The clinically comparable estimated effective doses per patient per acquisition method are displayed in Fig. 3. The mean of the effective dose for the clinical method was 32.2±5.4 mSv, the gating method was 37.0±8.4 mSv, rescan method was 43.5±7.4 mSv, and oversampling method was 64.8±11.8 mSv. All experimental 4D CT acquisition methods, if applied clinically, would impart higher effective doses than the standard clinical 4D CT.

### C. Statistical analysis

**Figure 4:**
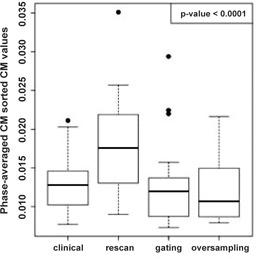
Boxplot of phase‐averaged CM values for each CM‐sorted acquisition method.

**Figure 5:**
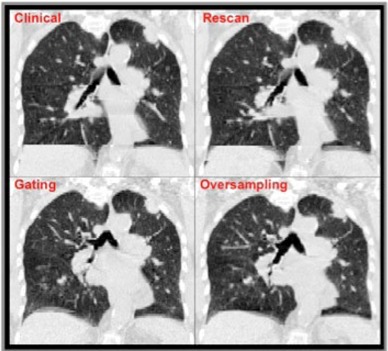
CM‐sorted sample coronal views.

Significant differences were evident among the acquisition methods test (p<0.0001, F‐test; Fig. 4). The mean CM values for the RM indicated an increase in artifact presence from the clinical acquisition (37%; p<0.002), the GM (26%; p<0.0052), and the OM (31%; p<0.001). The data lacked evidence of a significant difference among clinical, GM, and OM acquisitions using CM sorting, though the OM acquisition achieved the lowest mean and median CM values. The OM resulted in a 27% reduction in artifact presence from the current 4D CT implementation of the clinical acquisition with phase sorting (95% confidence interval=34−20). An example of coronal views across acquisition methods is displayed in Fig. 5.

The estimated probability of breathing irregularities and corresponding 95% confidence intervals are as follows: clinical acquisition 0.295 (CI: 0.262–0.329), GM 0.281 (CI: 0.254–0.308), RM 0.261 (CI: 0.218–0.309), and OM 0.270 (CI: 0.245–0.297). The extent of overlap in the confidence intervals precludes significant differences among any acquisition.

## IV. DISCUSSION

The OM achieved the highest significant artifact reduction of 27% compared to the current 4D CT implementation of the clinical acquisition with phase sorting, and contained the lowest artifact presence among CM‐sorted acquisitions. The OM contained the highest amount of data, yielding more alternative image options for phase binning than all other acquisition methods. The implementation was the simplest and most reproducible of all experimental acquisition methods, but it delivered the highest effective dose and had the longest scan duration due to the increase in cine duration necessary to capture at least 2 cycles of breathing data per couch position. Also, to implement the image limit change necessary for the OM, the CT scanner had to be restarted before each patient exam, which took approximately 8 min. However, if the OM were implemented clinically and the increased image limit was always available, the added time needed would not be an issue. Because CM‐sorted oversampling achieved the highest artifact reduction from the clinical standard and because the OM has a simple and reproducible implementation, we consider it to be the best acquisition/sorting combination among those tested.

The GM artifact reduction was not significantly lowered from the clinical acquisition and roughly equal in image quality to the clinical acquisition. This method suffered from visual breathing irregularity detection and lack of automatic X‐ray beam control. During visual irregularity detection, the last few breathing cycles behind the current breathing state only are visible on the respiratory trace, reducing the data that are integrated into the decision to stop the X‐ray beam. The manual X‐ray beam stop includes a time lag between verbal indications to the CT operator and the ensuing manual operation. These factors led to a mean 54.6% of breathing irregularities that remained during image acquisition, a mean 26.7% reduction in acquired breathing irregularities from the clinical acquisition. Although we were unable to exclude about half the irregularities occurring over the scan extent, we did reduce the irregularities from the clinical acquisition by 27%; we expected a larger artifact reduction in the GM from the clinical acquisition with this magnitude irregularity reduction. This could be from a lack in RPM correlation with internal anatomy or from the lack of a more robust irregularity quantification. The insignificant differences found in breathing irregularities between acquisitions may also suggest further development in the irregularity quantification is needed. The GM delivered the lowest overall effective dose among experimental methods, but was still higher than the clinical effective dose, due to the couch repetition inherent to the manual gating process.

The RM contained the highest artifact presence across all acquisitions and was significantly poorer in image quality. The RM suffered from combining two independent datasets, and from not gating irregularities in the reacquired images. The mean percentage of irregular breathing phases that occurred during the RM image reacquisition was 28.4%. To evaluate the anatomical shifts present in the RM, the T0% phase of the RM was fused with the T0% phase of the clinical 4D CT for each patient. One of 18 patients experienced a significant bone shift between the clinical 4D CT and RM, but most anatomical shifts were internal. The time between the beginning of the clinical acquisition and the RM was at least 5 min due to postprocessing and manual addition of specific couch positions, leading to these large anatomic shifts. Image segments were repeated without bias to scan location, resulting in a mean of 37.8% of the reacquired extent occurring in lung, with as few as one couch position repeated in the lung.

Our study focus was to explore alternative acquisition techniques as opposed to retrospective techniques to reduce 4D artifacts, as these are lacking in the literature.^22^, ^24^, ^36^, ^37^, ^38^ We performed these acquisitions on a relatively large sample of thoracic patients, without the use of simulations or phantoms,^23^, ^28^, ^39^ which may not truly represent breathing and anatomic changes present in actual patients. Artifacts were evaluated based on quantitative assessments of the images, relying on validation of the metric^29^, ^40^ and general agreement with visual observation. The metric does not correlate exactly to visual assessment, but provided a consistent method to evaluating scans in a relative fashion. This study focused on cine 4D CT, as that implementation of 4D CT was available at our institution, though any of the experimental acquisition methods could apply to helical 4D CT in principle.

Another weakness in this study is that each patient remained on the CT table in treatment position for at least 20 min longer than for routine clinical scans while the experimental scans were acquired. Patients frequently became tired or uncomfortable with longer scan times and the most promising acquisition methods were performed during the patient's worst state. Therefore, we expect scan quality to improve further if these methods are performed closer to when the patient first lies down on the table.

## V. CONCLUSIONS

Artifact presence in the clinical 4D CT acquisition was compared to three experimental acquisition methods: data oversampling, beam gating breathing irregularities, and rescanning clinical scan areas acquired during irregular breathing. Each was postprocessed by an alternative sorting method that optimizes a correlation‐based artifact metric, and the clinical acquisition was additionally postprocessed by the current standard, phase sorting. The oversampling method attained the highest statistically significant artifact reduction from the current 4D CT implementation and was the simplest and most reproducible to implement. The rescan method attained the highest statistically significant artifact presence, compared to all other acquisition methods.

## ACKNOWLEDGMENTS

We thank the thoracic radiation oncology physicians for allowing us to enroll their patients in this study, as well as the therapists and physics assistants for their assistance and patience. We also thank David Leos for his accommodation with patient recruitment, and many thanks to the participating patents. Financial support for this research was provided by the NIH Director's New Innovator Award DP2OD007044. Richard Castillo was partially supported by an NIH Research Scientist Development Award K01CA181292.
